# Synergy in Efficacy of *Artemisia sieversiana* Crude Extract and *Metarhizium anisopliae* on Resistant *Oedaleus asiaticus*

**DOI:** 10.3389/fphys.2021.642893

**Published:** 2021-03-22

**Authors:** Shuang Li, Chaomin Xu, Guilin Du, Guangjun Wang, Xiongbing Tu, Zehua Zhang

**Affiliations:** ^1^State Key Laboratory of Biology of Plant Diseases and Insect Pests, Institute of Plant Protection, Chinese Academy of Agricultural Sciences, Beijing, China; ^2^Scientific Observation and Experimental Station of Pests in Xilin Gol Rangeland, Institute of Plant Protection, Chinese Academy of Agricultural Sciences, Xilinhot, China; ^3^National Animal Husbandry Service, Ministry of Agriculture and Rural Affairs, Beijing, China

**Keywords:** *Artemisia*, *Metarhizium anisopliae*, *Oedaleus asiaticus*, synergetic control, biopesticide

## Abstract

In order to explore the synergistic control effect of crude extracts of *Artemisia sieversiana* and *Metarhizium anisopliae* on *Oedaleus asiaticus*, we used different doses of *M. anisopliae* and crude extracts of *A. sieversiana* singly and in combination, to determine their toxicities to fourth instar *O. asiaticus*. The results showed that the combination of 10% crude extract of *A. sieversiana* with 10^7^ and 10^8^ spores/g *M. anisopliae* concentrations and the combination of 20% crude extract of *A. sieversiana* with 10^7^ and 10^8^ spores/g *M. anisopliae* concentrations had significant effects on the mortality, body weight gain, body length gain, growth rate, and overall performance of *O. asiaticus* than those of the crude extract of *A. sieversiana* and *M. anisopliae* alone. Among them, the 20% *A. sieversiana* crude extract mixed with 10^8^ spores/g *M. anisopliae* and 10% *A. sieversiana* crude extract combined with 10^7^ spores/g *M. anisopliae*, had the best control efficacy. In order to clarify the biochemical mechanism underlying the immune responses of *O. asiaticus* to the pesticide treatments, we monitored the activities of four enzymes: superoxidase dismutase (SOD), peroxidase (POD), catalase (CAT), and polyphenol oxidase (PPO). The results showed that the activities of three enzymes (SOD, CAT, and PPO) were significantly increased from the treatment with the combination of *M. anisopliae* mixed with crude extract of *A. sieversiana*. Interestingly, compared to the crude extract, the combination treatment did not significantly induce the expression of POD enzyme activity, which may be a biochemical factor for increasing the control effect of the combination treatment. Our results showed that the combination treatment had synergistic and antagonistic effects on host mortality, growth, development, and enzyme activities in *O. asiaticus*.

## Introduction

As important biological control agents, botanical pesticides have received extensive research attention due to characteristics such as their low toxicities, easy degradation, and miscibility with other pesticides and against which insects are yet to develop resistance (Dubey et al., 2010). Crude plant extracts, the most notable of botanical pesticides, contain secondary metabolites such as alkaloids, terpenoids, phenols (mainly including flavonoids), tannins, lignin, nitrogenous organic compounds, etc., which have shown to be effective against insects (Wink, 1988). They have been extracted from plants species such as *Melilotus suavcolen*, *Peganum harmala* (zygophylllaceae), *Ajuga iva* (labiateae), *Aristolochia baetica* (Aristolochiaceae), *Raphanus raphanistrum* (Brassicaceae), etc. (Jbilou et al., 2006). These secondary metabolites are chemical compounds which have evolved in plants, to have specialized functions such as adaptation, survival of competition, defense against herbivorous insects, etc. They deter herbivorous insects from feeding on plants by poisoning them or interfering with digestion and the absorption of nutrients in herbivorous insects (Rattan, 2010). For instance, flavonoids can inhibit many insect species from feeding and oviposition (Simmonds, 2001). In addition, these substances also induce a rise in the detoxification mechanism of foreign compounds in insects (Bi and Felton, 1995). They can also stimulate and induce insects to feed or to produce sex hormones. Thus, they have mutually beneficial and co-evolutionary relationships (Kergoat et al., 2017).

China is rich in plant resources. There are more than 220 plant species that can be used as botanical insecticides. Compositae, one of the plant families which accounts for more than 20% of these species, is characterized by the most complex and diverse chemical compositions (Shang et al., 1997). *Artemisia*, a dominant genus in grasslands in Inner Mongolia, has shown promising insecticidal activities. In recent years, it has increasingly become one of the common plants, with the intensification of degradation of the grasslands in Inner Mongolia. The main chemical components of *Artemisia* are flavonoids, terpenoids, sesquiterpene lactones, cyanogenic glycides, flavonal, coumarins, etc. (Vander et al., 2008). Extensive studies have been conducted on the insecticidal activity of *Artemisia* plants (da Silva et al., 2005). For example, the crude extract of Anthemideae was shown to have strong anti-feedant properties against *Tribolium castaneum* Herbst, *Tetranychus cinnabarinus*, and *Maruca testulalis*. Also, *Artemisia mongolica*, *Artemisia lavandulaefolia*, and *Acacia vestita* showed excellent insecticidal effects against *Sitophilus zeamais* (Wu, 2015).

*Artemisia sieversiana*, a typical Artemisia plant, also known as Artemisia, *A. alba*, *A. scoparia*, etc., is one of the dominant species in grasslands in China. It is widely distributed in India, Pakistan, Afghanistan, Russia, Mongolia, and China’s Heilongjiang, Shanxi, Hebei, Ningxia, Gansu, Xinjiang, Inner Mongolia, and other regions (Cui et al., 2020). *A. sieversiana* contains a variety of insecticidal and antibacterial chemical components, such as flavonoids, lignins, sesquiterpenes, and volatile oils (Liu et al., 2010; Cui et al., 2020). In addition, its essential oil was shown to have fumigation and killing activity on *Callosobruchus chinensis*, grubs, and other insects (Ayub et al., 2019). Further, the ethanol extract of *A. sieversiana* was shown to have significantly inhibited and killed the growth of human colon cancer cells (HT-29, HCT-15, and Colo-205; Tang et al., 2015).

Entomopathogenic fungi are another group of important biological control agents with low environmental risk, used in pest management. Insect pests are not easily resistant to entomopathogenic fungi. They have great potential for pest control in integrated pest management programs (Blanford et al., 2005; Sabbour and Sahab, 2005; Zimmermann, 2007). *Metarhizium anisopliae*, one of such entomopathogenic fungal species, is widely used globally, and has been studied for more than 100 years in China. It has been used on more than 200 insect species and has shown significant control effects against the larvae of Curculio chinensis Chevrolat, *Monochamus alternatus*, Polyphylla laticollis Lewis, Locusta migratoria manilensis, *Plutella xylostella*, *O. asiaticus*, etc. (Peng et al., 2008; Kaaya et al., 2011).

*M. anisopliae* infects insects through the insect cuticle or when insects feed (stomach poison; Mannino et al. 2019). *M. anisopliae* directly enters the midgut through the feeding of insects, and the mycelium propagates rapidly in the midgut cells, resulting in the loss of cellular functions, directly affecting cellular metabolism, and ultimately destroying the midgut tissue. These result in the death of insects. Mortality rates of insects from midgut infections are significantly higher than that from cuticle infections. *M. anisopliae* infections are very easy to spread in their colonies. However, the long lethal time of fungal insecticides lowers their efficacy (MeCoy, 1981; Dimbi et al., 2009).

All groups of insecticides, including botanical insecticides, fungicides, and chemical insecticides, etc., have advantages and disadvantages (Marican and Durán-Lara, 2018). For example, botanical insecticides are environment friendly, but also have unstable formulations, easy photodegradation and their active ingredients are difficult to extract (Pavela, 2016). Insects do not easily develop resistance against fungal insecticides, but their lethal times are longer, and their prevention and control of pest outbreaks is slower. Chemical insecticides show rapid effectiveness and good control effects, but their excessive usage generates residues which pollute the environment and also have negative impact on food safety for humans and animals (Sabarwal et al., 2018). However, the use of a combination of these insecticides can effectively solve these challenges by producing a synergistic efficacy to achieve better control of insect pest. It can also solve the challenge of excessive use of chemical agents (Gibson et al., 2014). For example, the use of a combination of *M. anisopliae* V275 and imidacloprid or fipronil had a significantly higher control effect on *Otiorhynchus sulcatus* than that from each insecticide alone (Shah et al., 2007). Also, neem oil effectively maintained the toxicity of the entomopathogenic fungus *M. anisopliae*, protected it from UV radiation, and increased the control effect on *Aedes aegypti* larvae (Paula et al., 2019). The use of a combination of the insecticide chlorantraniliprole and *M. anisopliae* showed excellent synergistic control on locusts, scarabaeidea, Empoasca pirisuga Matumura, rice planthoppers, and other agricultural pest (Farenhorst et al., 2010; Jia et al., 2016).

*Oedaleus decorus asiaticus* (Bey-Bienko), is a major pest in the grasslands of northern China. In recent years, its frequent large-scale outbreaks are partly responsible for the degradation and desertification of grasslands in Inner Mongolia, in China (Zhang et al., 2013). At present, the main control of *O. asiaticus* is based on biological control methods such as the use of poxvirus, *M. anisopliae*, *Beauveria bassiana*, and the introduction of insect natural enemies. Among them, *M. anisopliae* has shown a very significant control effect on *O. asiaticus*. It has the advantage of being green, safe, and has long duration, but has a slow effect on insects. Quercetin (a kind of flavonoids) was shown to reduce the survival rate, inhibit the growth and development of *O. asiaticus* (Cui et al., 2019). The crude extract of *A. sieversiana* contains flavonoids, which has been shown to have a toxic effect on insects, but its efficacy lasted for a short period of time. For practical applications, the simultaneous use of fungi and chemical agents for insect pest control, may result in higher mortality than from the use of each alone (Duarte et al., 2016; Jia et al., 2016). Therefore, we evaluated the synergetic control effect of the crude extract of *A. sieversiana* and *M. anisopliae* on *O. asiaticus* in this study, to provide a new direction for the research and development of new biological agents for the effective control of locust or grasshopper pest.

## Materials and Methods

### Experimental Materials

#### Test Insects

*O. asiaticus* were caught from the wild, in the grasslands of Xilinhot City, Inner Mongolia, China. They were reared on their host plant, *Stipa krylovii*, in a cage (1 m × 1 m × 1 m) at room temperature (about 27°C) for 1 week.

#### Test Plant

*A. sieversiana* was collected from the Scientific Observing and Experimental Station of Pests in Xilingol Rangeland, Ministry of Agriculture, P.R. China (E116°36'', N43.57'12''), located in the Xilingol League, Inner Mongolia, northeast China.

#### Test Strain

*M. anisopliae* IMI330-189 was provided by the Grassland Pest Group, Institute of Plant Protection, Chinese Academy of Agricultural Sciences (IPPCAAS), which was introduced from the International Centre for Biological Control, United Kingdom, and preserved in the IPPCAAS.

#### Test Instruments

Insect net (mesh diameter: 5 mm, mesh bag diameter: 40 cm, and mesh length: 80–130 cm), insect cage (1 m × 1 m × 1 m), insect rearing frame (26 × 13 × 7 cm), glass cover plate (30 × 50 × 0.3 cm), conical flask (100 ml), test tube (10 ml), petri dish, writing brush, stirring rod, glass beads (dm = 3 mm), hemocytometer (25 × 16 mm), microscope (OLYMPUS-SZX16), oscillator MX-F, oven DHG-9123A, autoclave YXQ-LS-SII, and electronic balance PL-403.

#### Test Agent

Liquid nitrogen, distilled water, soil temperature 80 (dosage was 0.001), vegetable oil, and carrier (wheat bran).

### Method

#### Preparation of Crude Plant Extract

The crude plant extract was extracted with water and two concentrations (10 and 20%) of the extracts were prepared (Table 1). Briefly, the fresh plant materials of *A. sieversiana* were cut into small pieces of about 1 cm and put into a conical flask. Distilled water was added and then autoclaved at 120°C for 90 min. They were taken out after heating and cooled to room temperature to filter plant residues.

**Table 1:** Proportion of components in the different concentrations of the prepared crude extract from *Artemisia sieversiana*.

Concentration (%)	Amount of distilled water (g)	Amount of *A. sieversiana* (g)
10	500	50 g
20	500	100 g

### Toxicological Tests

#### Separate Treatments With *A. sieversiana* Extract or *M. anisopliae*

Healthy 4th-instar *O. asiaticus* were selected from the insect cage in the laboratory, and raised in a 26 × 13 × 7 cm^3^ breeding frame with a glass cover (30 × 50 × 0.3 cm). Each cage contained 10 females and 10 males (20 heads in each frame). One frame served as a replicate, and five repetitions were treated as a group. In the *A. sieversiana* extract treatment, the 10 and 20% *A. sieversiana* extracts were spread separately on leaves of *S. krylovii* with a brush and fed to *O. asiaticus* in the cages. Leaves on which distilled water was spread, served as the control. The experiment lasted for 7 days and data on mortality were recorded.

In the *M. anisopliae* treatment, 1 g each of the three concentrations of *M. anisopliae* (10^6^, 10^7^, and 10^8^ spores/g) was weighed and separately mixed with a carrier (10 g vegetable oil and 100 g wheat bran) as poison bait. The mixture was divided into five petri dishes (dm = 7 cm) and placed in the insect rearing frame containing 20 healthy 4th instar *O. asiaticus* which had been prior fed with fresh S. kirnii for 24 h. The experiment lasted for 7 days and data on mortality were recorded.

#### Treatment With the Combination of *A. sieversiana* Extract + *M. anisopliae*

In this treatment, mixtures which were made up of each concentration of *A. sieversiana* extract and each concentration of *M. anisopliae* were prepared (Table 2). These combinations were then added to a mixture made up of 10 g vegetable oil mixed into 100 g wheat bran. They were mixed evenly, and then dispensed into glass culture dishes to feed *O. asiaticus* in the cages. After feeding for 24 h, the diet was changed to fresh *S. kirnii* for continuous feeding for 7 days. Data on mortality were recorded during this period.

**Table 2:** Mixed formula of crude extract of *A. sieversiana* and *Metarhizium anisopliae*.

Order number	Concentration of crude extract of *A. sieversiana* (%)	*M. anisopliae* concentration (spores/g)
1	10	10^6^
2	10	10^7^
3	10	10^8^
4	20	10^6^
5	20	10^7^
6	20	10^8^

### Determination of Body Weight and Body Length of *O. asiaticus*

Female and male 4th instar *O. asiaticus* caught from the Xilinguole grassland were used to determine the effects of the treatments on body weight. We randomly selected 30 healthy 4th females and 4th males, respectively, from the captured 4th instar *O. asiaticus* to detect their initial body weight and body length. We use a vernier caliper to measure these selected females and males, and then put them in the oven at 90°C for 24 h after which the dry weights were measured using an electronic balance.

We also selected enough healthy 4th instar females and 4th instar males from the captured *O. asiaticus* for experiments. We conducted the experiment twice, each with five biological replicates, each cage contained 10 females and 10 males (20 heads in each frame). The survival and developmental time of *O. asiatica* in the cages were recorded every day to ensure that there were enough males and females for body weight and body length measurement at the end of the experiment. Dead individuals were removed in time when we change fresh *S. krylovii*. At the end of 7 days, the body lengths of 30 locusts were measured using the vernier caliper. They were then dried in the oven at 90°C for 24 h after which the dry weights were measured using an electronic balance.

### Determination of Activities of Antioxidant Enzymes in *O. asiaticus*

The effect of the pesticide treatments on activities of four antioxidant enzymes [superoxidase dismutase (SOD), peroxidase (POD), catalase (CAT), and polyphenol oxidase (PPO)] in *O. asiaticus* were measured. To extract crude enzyme solutions, about 0.1 g of insect tissue was weighed, to which 1 ml of an extraction solution was added (purchased from Suzhou Keming Biotechnology Co., Ltd.). They were then placed in an ice bath for homogenization. The supernatant was then centrifuged at 8000 *g* at 4°C for 10 min and placed on ice for the measurement of enzyme activities with detection kits of each antioxidant enzymes (Suzhou Keming Biotechnology Co., Ltd.). Preparation of the test samples followed the manufacturer’s instructions. The absorbance wavelength of each enzyme was measured on a microplate reader (DNM-9602G).

For POD, the wavelength was adjusted to 470 nm, and that of distilled water adjusted to zero. The initial absorbance value, A1, was recorded at 470 nm at 1 min and that of A2 at 2 min. The final absorbance value was given by ΔA = A2−A1. For CAT, the wavelength was adjusted to 240 nm, and that of distilled water was adjusted to zero. The initial absorbance value A1 was recorded at 240 nm and the 2nd absorbance value A2, was recorded after 1 min. The final absorbance value was given by ΔA = A1−A2. For PPO, the wavelength was adjusted to 525 nm, and that of distilled water was adjusted to zero. The absorbance of the determination tube and reference tube was detected at 525 nm. The final absorbance value was given by ΔA = A measured-A control. For SOD, the absorbance was measured at 560 nm. The final absorbance value was given by the formula, Inhibition percentage = (A control tube-control tube) ÷ A control tube × 100%.

### Data Analysis

(1) The mortality rate (%) was calculated as the ratio of the number of *O. asiaticus* deaths in each frame to the number of *O. asiaticus* in the initial 4th instar (*N* = 20). (2) Weight gain (increased body dry mass, mg) = dry weight of *O. asiaticus* after 7 days (mg) − 4th instar *O. asiaticus* weight (mg). (3) Increase in body length (mm) = 7 days later *O. asiaticus* length (mm) − the length of the first 4th instar *O. asiaticus* (mm). (4) The developmental period was the experimental period of 7 days. (5) Growth rate (mg/day) = weight gain ÷ developmental duration. (6) Overall performance = survival rate × growth rate (Cease et al., 2012; Cui et al., 2019).

The activities of the enzymes were determined using the following formulae;

Paragraph text.

(1) $$\begin{array}{l} \mathrm{POD}\;\left(U/g_{\mathrm{fresh weight}}\right)=\Delta A\times V_{\mathrm{total reaction}}/\\ \;\;\;\;\;\;\;\;\;\;\;\;\;\;\;\;\;\;\;\;\;\;\;\;\;\;\;\;\;\;\;\;\;\;\;\;\;\;\;\;\;\;\;\;\;\;\;\;\;\;\;\;\;\;\;\;\;\;\;\;\;\;\left(500\times V_{\mathrm{sample}}/V_{\mathrm{sample total}}\right)/0.005/T\\ \;\;\;\;\;\;\;\;\;\;\;\;\;\;\;\;\;\;\;\;\;\;\;\;\;\;\;\;\;\;\;\;\;\;\;\;\;\;\;\;\;\;\;\;\;\;\;\;\;\;\;\;\;\;\;\;=4000\times \Delta A/W \end{array}$$

(2) $$\begin{array}{l} \mathrm{CAT}\;\left(U/g_{\mathrm{fresh weight}}\right)=\left[\Delta A\times V_{\mathrm{total reaction}}/\left(\mu \times d\right)\times 109\right]/\\ \;\;\;\;\;\;\;\;\;\;\;\;\;\;\;\;\;\;\;\;\;\;\;\;\;\;\;\;\;\;\;\;\;\;\;\;\;\;\;\;\;\;\;\;\;\;\;\;\;\;\;\;\;\;\;\;\;\;\;\;\left(W\times V_{\mathrm{sample}}/V_{\mathrm{sample total}}\right)/T\\ \;\;\;\;\;\;\;\;\;\;\;\;\;\;\;\;\;\;\;\;\;\;\;\;\;\;\;\;\;\;\;\;\;\;\;\;\;\;\;\;\;\;\;\;\;\;\;\;\;\;\;\;\;\;\;=918\times ”\;A/W \end{array}$$

(3) $$\begin{array}{l} \mathrm{PPO}\;\left(U/g_{\mathrm{fresh weight}}\right)=\Delta A\times V_{\mathrm{total reaction}}/\\ \;\;\;\;\;\;\;\;\;\;\;\;\;\;\;\;\;\;\;\;\;\;\;\;\;\;\;\;\;\;\;\;\;\;\;\;\;\;\;\;\;\;\;\;\;\;\;\;\;\;\;\;\;\;\;\;\;\;\;\;\left(W\times V_{\mathrm{sample}}/V_{\mathrm{sample total}}\right)/0.005/T\\ \;\;\;\;\;\;\;\;\;\;\;\;\;\;\;\;\;\;\;\;\;\;\;\;\;\;\;\;\;\;\;\;\;\;\;\;\;\;\;\;\;\;\;\;\;\;\;\;\;\;\;\;\;\;\;=120\times \Delta A/W \end{array}$$

(4) $$\begin{array}{l} \mathrm{SOD}\;\mathrm{activity}\;\\ \left(U/g_{\mathrm{fresh weight}}\right)=\left[\begin{array}{l} \mathrm{inhibition percentage}÷\\ \left(1-\mathrm{inhibition percentage}\right)\times \\ V_{\mathrm{total}} \end{array}\right]\\ \;\;\;\;\;\;\;\;\;\;\;\;\;\;\;\;\;\;\;\;\;\;\;\;\;\;\;\;\;\;\;\;\;\;\;\;\;\;\;\;÷\;\;\left(W\times V_{\mathrm{sample}}÷V_{\mathrm{sample total}}\right)\\ \;\;\;\;\;\;\;\;\;\;\;\;\;\;\;\;\;\;\;\;\;\;\;\;\;\;\;\;\;\;\;\;\;\;\;\;\;\;\;\;=11.11\times \mathrm{inhibition percentage}÷\\ \;\;\;\;\;\;\;\;\;\;\;\;\;\;\;\;\;\;\;\;\;\;\;\;\;\;\;\;\;\;\;\;\;\;\;\;\;\;\;\;\;\;\;\;\;\left(1-\mathrm{inhibition percentage}\right)÷W \end{array}$$

(V_total reaction_: total volume of reaction; V_sample_: volume of added sample; V_sample total_: volume of added extract; T: reaction time; W: sample mass; ε: molar extinction coefficient of H_2_O_2_, 4.36 × 104 L/mol/cm)

The experimental data were all analyzed using Excel 2010, the means were evaluated *via* the one-way ANOVAs at the 5% significance level, and differences among means were compared by using the Turkey at *p* < 0.05 (Jia et al., 2016). And Prism6 was used for making the figure, and the error bars is the SE.

## Results

### Effects of the 10% *A. Sieversiana* (10% AS) Extract, Different Concentrations of *M. anisopliae* and Their Combinations on the Growth and Development of *O. asiatica*

#### Effect on Weight

**Figure 1:**
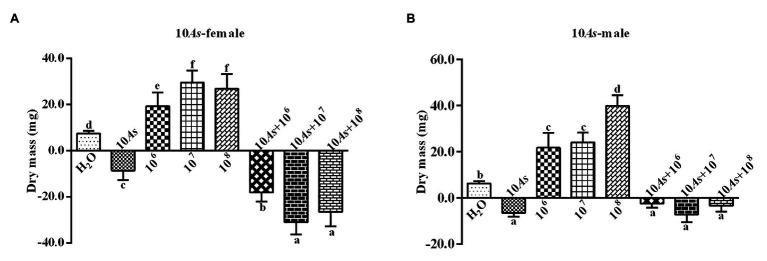
Body weight gain and loss of **(A)** female and **(B)** male 4th instar *Oedaleus asiatica* from treatments with 10% *A. sieversiana* (10% As) crude extract, different concentrations of *M. anisopliae* and their combinations. Different lowercase letters indicate significant differences in the growth rate of the *O. asiatica* in the same year.

The 10% crude extract of *A. sieversiana* had significant inhibitory effect on the body weight of female (Figure 1A) and male 4th instar (Figure 1B) *O. asiatica*. However, the different concentrations (10^6^, 10^7^, and 10^8^ spores/g) of *M. anisopliae* promoted weight gain in female (Figure 1A) and male 4th instar (Figure 1B) *O. asiatica*. Among them, the 10^7^ and 10^8^ spores/g *M. anisopliae* concentrations had the strongest effect on the weight gain of female (29.5 ± 1.63 g) and male *O. asiatica* (26.9 ± 2.00 g), respectively. The combined treatment of 10% crude extract of *A. sieversiana* and each of the different concentration of *M. anisopliae*, had a significant inhibitory effect on the weight gain of *O. asiatica*. Among them, the combination of 10% *A. sieversiana* crude extract + 10^7^ spores/g of *M. anisopliae* had the most significant inhibitory effect on the weight gain of the female (Figure 1A) and male 4th instar (Figure 1B) *O. asiatica* (*F* = 233.829, DF = 7, *p* = 0), with the weight gain of 129.73 ± 1.97 g and −7.2 ± 1.07, respectively.

#### Effects on Body Length

**Figure 2:**
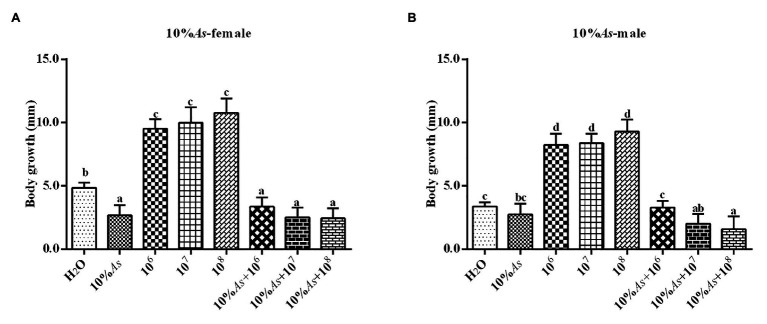
Increase in body length of the **(A)** female and **(B)** male 4th instar *O. asiatica* from the treatment with 10% *A. sieversiana* (10% As) crude extract, different concentrations of *M. anisopliae* and their combinations. Different lowercase letters indicate significant differences in the growth rate of the *O. asiatica* in the same year.

The 10% crude extract of *A. sieversiana* and *M. anisopliae* alone or in combination had effects on the body length of female (Figure 2A) and male (Figure 2B) *O. asiatica*. The 10% crude extract of *A. sieversiana* had an inhibitory effect on the growth of the body length of the *O. asiatica*. The different concentration (10^6^, 10^7^, and 10^8^ spores/g) of *M. anisopliae* all increased the body length of the *O. asiatica*, of which the 10^8^ spores/g *M. anisopliae* concentration had the most obvious effect on male (10.78 ± 0.36 mm) and female (9.28 ± 0.31 mm) *O. asiatica*. The combined treatment of 10% crude extract of *A. sieversiana* and the different concentrations of *M. anisopliae* (10^6^, 10^7^, and 10^8^ spores/g) had an inhibitory effect on the body length of *O. asiatica*. The combined treatment of 10% of *A. sieversiana* crude extract + 10^8^ spores/g of *M. anisopliae*, had the most significant inhibitory effect on the increase in body length of *O. asiatica* (Female: 3.66 ± 0.23 mm; male: 2.87 ± 0.18 mm).

#### Effects on Growth Rate

**Figure 3:**
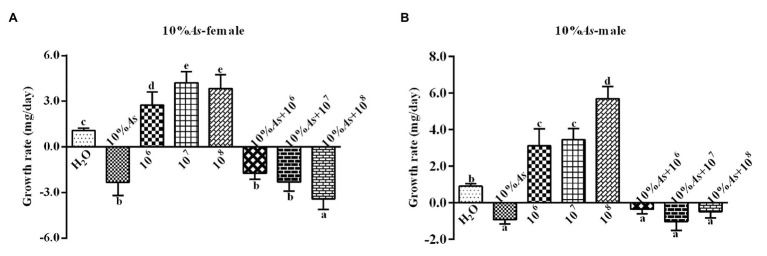
Growth rates of **(A)** female and **(B)** male 4th instar *O. asiatica* from the treatments with 10% *A. sieversiana* (10% As) crude extract, different concentrations of *M. anisopliae* and their combinations. Different lowercase letters indicate significant differences in the growth rate of the *O. asiatica* in the same year.

The 10% crude extract of *A. sieversiana* and *M. anisopliae* alone or in combination had effects on the growth rate of female (Figure 3A) and male (Figure 3B) *O. asiatica*. The growth rate of *O. asiatica* was significantly inhibited by the 10% crude extract of *A. sieversiana*, However, all the concentrations of *M. anisopliae* (10^6^,10^7^, and 10^8^ spores/g) promoted the growth rate of female and male grasshoppers. For female, the 10^7^ spores/g concentration had the highest effect on growth rate. For male, the 10^8^ spores/g concentration had the highest effect. The results showed that the combination of the 10% crude extract of *A. sieversiana* and the different concentrations of *M. anisopliae* (10^6^, 10^7^, and 10^8^ spores/g) had significant inhibitory effect on the growth rate of *O. asiatica*. The combination of 10% crude extract + 10^8^ spores/g *M. anisopliae* and 10% crude extract + 10^7^ spores/g *M. anisopliae* had the most significant inhibitory effect on the growth rate of female and male *O. asiatica*, respectively.

#### Effects on Overall Performance

**Figure 4:**
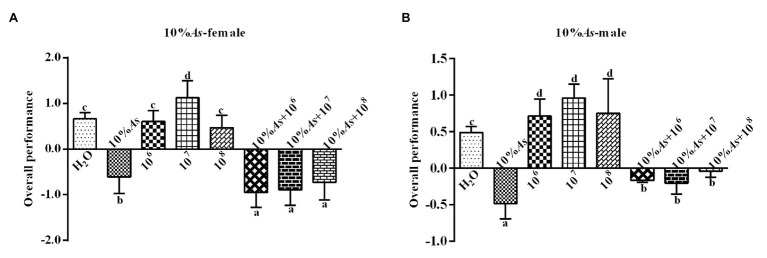
Overall performance of **(A)** female and **(B)** male 4th instar *O. asiatica* from the treatments with 10% *A. sieversiana* (20% As) crude extract, different concentrations of *M. anisopliae* and their combinations. Different lowercase letters indicate significant differences in the overall performance of the *O. asiatica* in the same year.

The 10% *A. sieversiana* crude extract and *M. anisopliae* alone or in combination had effect on the overall performance of female (Figure 4A) and male (Figure 4B) *O. asiatica*. The 10% crude extract of *A. sieversiana* had an extremely significant effect on the overall performance. All concentrations of *M. anisopliae* (10^6^, 10^7^, and 10^8^ spores/g) promoted the growth and development of *O. asiatica*. Among them, the 10^7^ spores/g *M. anisopliae* concentration had the most significant effect on the growth and development of male (1.13 ± 0.37) and female (0.96 ± 0.08) *O. asiatica*. The combined treatment of 10% crude extract of *A. sieversiana* + each of the concentration of *M. anisopliae* (10^6^, 10^7^, and 10^8^ spores/g), had an effection on the growth of *O. asiatica*. The combined treatment of 10% *A. sieversiana* crude extract + 10^6^ spores/g *M. anisopliae* and 10% crude extract of *A. sieversiana* + 10^7^ spores/g *M. anisopliae* had the best inhibition on the overall performance of female *O. asiatica* (−0.95 ± 0.15) and male *O. asiatica* (−0.21 ± 0.07), respectively.

### Effects of the 20% *A. sieversiana* (20% AS), Different Concentrations of *M. anisopliae* and Their Combinations on the Growth and Development of *O. asiatica*

#### Effects on Body Weight

**Figure 5:**
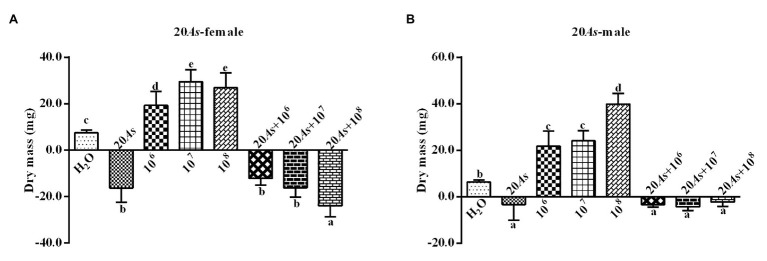
Weight gain and loss of the **(A)** female and **(B)** male 4th instar *O. asiatica* from the treatments with 20% *A. sieversiana* (20% As) crude extract, different concentrations of *M. anisopliae* and their combinations. Different lowercase letters indicate significant differences in the weight gain of the *O. asiatica* in the same year.

The 20% crude extract of *A. sieversiana* had a significant inhibitory effect on the body weight of female (Figure 5A) and male (Figure 5B) 4th instar *O. asiatica*. However, the different concentrations of *M. anisopliae* (10^6^, 10^7^, and 10^8^ spores/g) promoted weight gain in the female and male *O. asiatica*. Among them, the 10^7^ spores/g *M. anisopliae* concentration had the strongest effect on the weight gain of female *O. asiatica* (19.3 ± 1.88 mg). The combined treatment of 20% *A. sieversiana* crude extract and the different concentrations of *M. anisopliae* (10^6^, 10^7^, 10^8^ spores/g) had a significant inhibitory effect on the weight gain of the *O. asiatica*. Among them, the combined treatment of 20% crude extract of *A. sieversiana* + 10^8^ spores/g of *M. anisopliae* and 20% *A. sieversiana* crude extract + 10^7^ spores/g of *M. anisopliae* had the strongest inhibitory effect on the weight gain of the male (−23.9 ± 1.05 mg) and female (−4.3 ± 0.52 mg) *O. asiatica*, respectively.

#### Effects on Body Length

**Figure 6:**
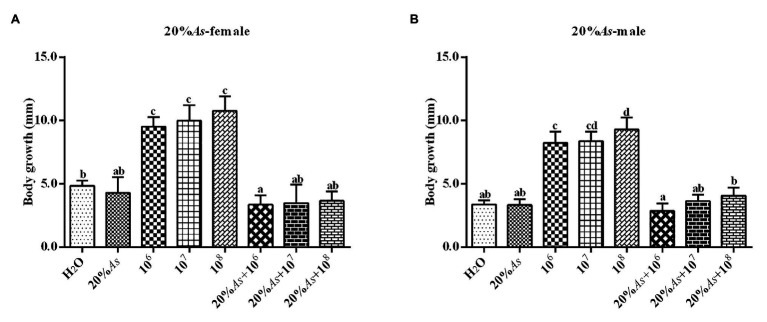
Increase in body length of **(A)** female and **(B)** male 4th instar *O. asiatica* from treatments with 20% *A. sieversiana* (20% As) crude extract, different concentrations of *M. anisopliae* and their combinations. Different lowercase letters indicate significant differences in the body length of the *O. asiatica* in the same year.

The 20% crude extract of *A. sieversiana* and *M. anisopliae* alone or in combination had effect on the body length of the female (Figure 6A) and male (Figure 6B) *O. asiatica*. The 20% crude extract of *A. sieversiana* had a relatively inhibitory effect on the body length of the *O. asiatica*. The different concentrations of *M. anisopliae* (10^6^, 10^7^, and 10^8^ spores/g) increased the body length of *O. asiatica*. Among them, the 10^8^ spores/g *M. anisopliae* concentration had the most significant effect on the body length. The combined treatment of 20% crude extract of *A. sieversiana* and different concentrations of *M. anisopliae* (10^6^, 10^7^, and 10^8^ spores/g) had an inhibitory effect on the body length of female and male *O. asiatica*. The combined treatment of 20% crude extract of *A. sieversiana* + 10^8^ spores/g *M. anisopliae* had the most significant inhibitory effect on the body length of *O. asiatica* (female 2.44 ± 2.52 mm; male: 1.58 ± 0.32 mm).

#### Effects on Growth Rate

**Figure 7:**
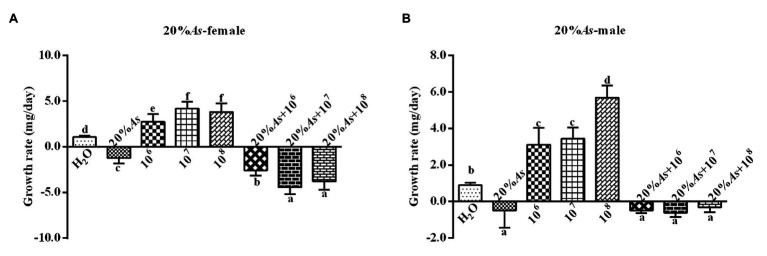
Growth rates of **(A)** female and **(B)** male 4th instar *O. asiatica*, from treatments with 20% *A. sieversiana* (20% As) crude extract, different concentrations of *M. anisopliae* and their combinations. Different lowercase letters indicate significant differences in the growth rate of the *O. asiatica* in the same year.

The 20% *A. sieversiana* crude extract and *M. anisopliae* alone or in combination had effects on the growth rate of female (Figure 7A) and male (Figure 7B) *O. asiatica*. The 20% crude extract of *A. sieversiana* had inhibitory effects on the growth rates of *O. asiatica*. The results also showed that the concentration of 10^7^ spores/g *M. anisopliae* and 10^8^ spores/g of *M. anisopliae* had a significant effect on the growth rate of female (*F* = 185.11, DF = 7, *p* = 0), and male (*F* = 161.59, DF = 7, *p* = 0) *O. asiatica*. The combination treatment of 20% crude extract + 10^8^ spores/g *M. anisopliae* had the most significant inhibitory effect on the growth rate of female (−3.41 ± 0.22 mg/d) and combination treatment of 20% crude extract + 10^7^ spores/g *M. anisopliae* had the most significant inhibitory effect on the growth rate of male (−0.61 ± −0.78 mg/d) *O. asiatica*.

#### Effects on Overall Performance

**Figure 8:**
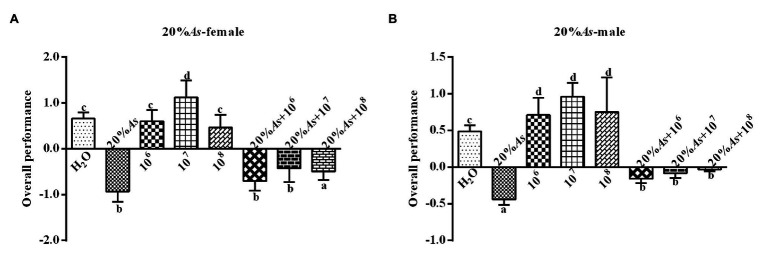
Overall performance of **(A)** female and **(B)** male 4th instar *O. asiatica* from treatments with 20% *A. sieversiana* (20% As) crude extract, different concentrations of *M. anisopliae* and their combinations. Different lowercase letters indicate significant differences in the overall performance of the *O. asiatica* in the same year.

The 20% *A. sieversiana* crude extract and *M. anisopliae* alone or in combination had effects on the overall performance of female (Figure 8A) and male (Figure 8B) *O. asiatica*. The 20% crude extract of *A. sieversiana* had an inhibition on the overall performance of female (−0.93 ± 0.10) and male (−0.44 ± 0.03). The 10^7^ spores/g *M. anisopliae* concentration had a promoting effect on the growth and development of female (1.13 ± 0.16) and male (0.96 ± 0.08) *O. asiatica* compared to the control. The combination of the 20% *A. sieversiana* crude extract and different concentration of *M. anisopliae* had a significant inhibitory effect on the overall performance of *O. asiatica*. Among them, the combination of 20% *A. sieversiana* crude extract + 10^6^ spores/g had the strongest inhibition on the overall performance of female (−0.71 ± 0.09; Figure 8A) and male (−0.16 ± 0.03) *O. asiatica* (Figure 8B).

### Effect of Crude Extracts of *A. sieversiana*, Different Concentrations of *M. anisopliae* and Their Combinations on the Mortality of *O. asiatica*

**Figure 9:**
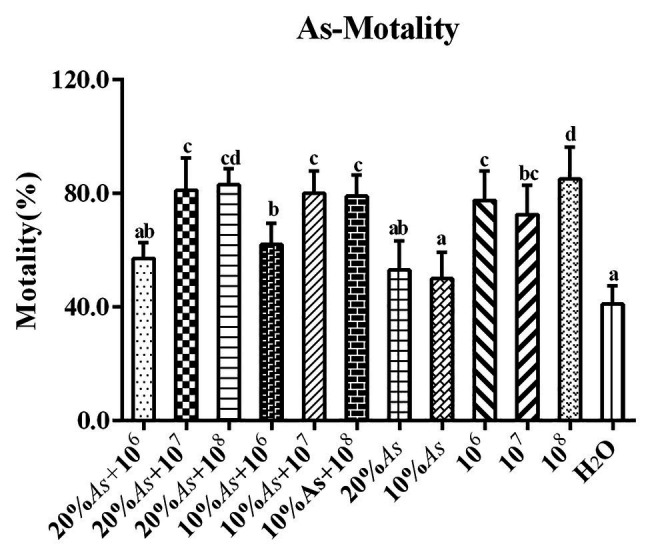
Mortalities of *O. asiatica* from treatments with 10 and 20% crude extract *A. sieversiana* and *M. anisopliae* alone or in combination. Different lowercase letters indicate significant differences in the mortality of the *O. asiatica* in the same year.

When *A. sieversiana* was used alone, the lethal effect of 20% crude extract was greater than that of 10% crude extract. The lethal effect of the different concentrations of *M. anisopliae* on *O. asiatica* followed a decreasing order as follows: 10^8^ spores/g > 10^6^ spores/g > 10^7^ spores/g, but there was no significant difference between the 10^6^ and 10^7^ spores/g concentration. The lethal effect of combined treatment on *O. asiatica* also followed a decreasing order as follows: 20% As + 10^8^ spores/g *M. anisopliae* > 20% As + 10^7^ spores/g *M. anisopliae* > 10% As + 10^7^ spores/g *M. anisopliae* > 10% As + 10^6^ spores/g *M. anisopliae* > 20% As + 10^6^ spores/g *M. anisopliae*. Among them, 10^8^ spores/g *M. anisopliae* had the best efficacy, resulting into a mortality rate of 85 ± 5.08%. The results showed that the combination of 10% crude extract of *A. sieversiana* + 10^7^ and 10^8^ spores/g *M. anisopliae*, the combination of 20% crude extract + 10^7^ and 10^8^ spores/g *M. anisopliae* had significantly better control effect on *O. asiatica* than that of using only crude extract of *A. sieversiana* (*F* = 14.23, DF = 11, *p* = 0; Figure 9).

### Effect of Crude Extracts of *A. sieversiana*, Different Concentrations of *M. anisopliae* and Their Combinations on the Activities of Antioxidant Enzymes in *O. asiatica*

**Figure 10:**
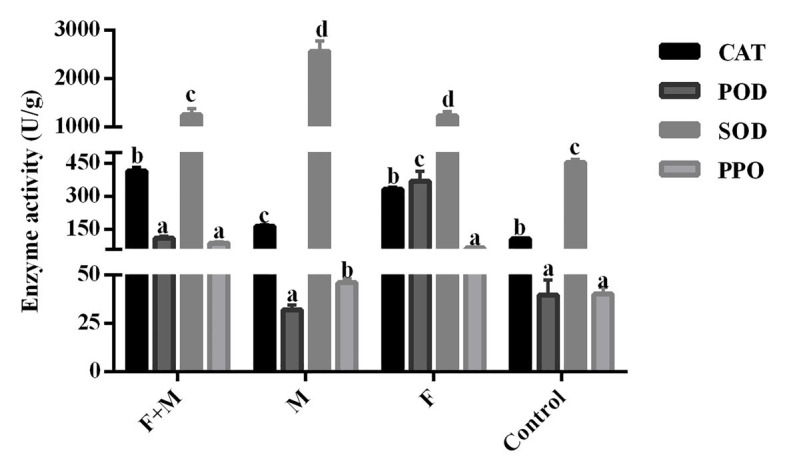
Activities of antioxidant enzymes in *O. asiatica* from the treatment with 10% crude extract *A. sieversiana* and *M. anisopliae* alone or their combined use. 1. F, *M. anisopliae* alone; M, crude extract of 10% *A. sieversiana* alone; F+M, combination of *M. anisopliae* and the 10% crude extract of *A. sieversiana*; Control, distilled water; CAT, catalase; SOD, superoxide dismutase; POD, peroxidase; PPO, polyphenol oxidase.

There was no significant difference in the lethal effect of the 20% As + 10^7^ spores/g *M. anisopliae* and 10% As + 10^7^ spores/g *M. anisopliae* on *O. asiatica* (Figure 9). Likewise the effect of the increase in concentration of *A. sieversiana* extracts, on the mortality of *O. asiatica* was not significantly different. Therefore, in this experiment, we tested the activities of antioxidant enzymes in *O. asiatica* from the following treatments: 10% *A. sieversiana* extract + 10^7^ spores/g *M. anisopliae*, 10% *A. sieversiana* extract alone, 10^7^ spores/g *M. anisopliae* alone, and distilled water (control). The activities of catalase (CAT; 414.94 ± 16.94 U/g) and polyphenol oxidase (PPO; 88.20 ± 5.49 U/g) were significantly increased by the 10% *A. sieversiana* extract + 10^7^ spores/g *M. anisopliae* (Figure 10). The activities of SOD and POD were also activated by this treatment. The highest activity of SOD (2564.10 ± 218.08 U/g) was recorded under the 10^7^ spores/g *M. anisopliae* treatment, and that of POD (368.40 ± 47.45 U/g) was recorded under the 10% *A. sieversiana* extract treatment (Figure 10).

## Discussion

The frequent large-scale outbreaks of *O. asiatica* in the grasslands of northern China, are partly responsible for the recent degradation of grasslands and desertification in Inner Mongolia in China. The entomopathogenic fungus *M. anisopliae*, a recognized non-polluting and non-toxic pesticide, has been widely studied for pest control and has shown potential as a chemical substitute for locust and grasshopper control (Gillespie et al., 2000; Peng et al., 2008). However, insect pathogenic fungi have shortcomings such as a longer time of attacking pest and unstable control effect (Pilz et al., 2009). In addition, external conditions such as temperature, humidity, density, and height of vegetation also affect the control efficacy of *M. anisopliae* on locust. In dense vegetation areas, the reduced infectivity of *M. anisopliae* conidia on the vegetation surface also reduces its efficacy (Hunter et al., 2001).

Therefore, the use of appropriate adjuvants can increase the persistence of fungi. Many entomopathogenic fungi are compatible with a range of chemical and natural insecticides (Santos et al., 2007; Pelizza et al., 2015). One possible candidate is crude plant extracts. For example, the combination of neem oil and *M. anisopliae* significantly increased the mortalities of *A. aegypti* and locusts. By this, the efficacy of this biological control agent was increased (Haroona et al., 2011; Paula et al., 2019). However, neem oil inhibited the vegetative growth of Beauveria bassiana and reduced the yield and viability of conidia (Depieri et al., 2005).

The secondary metabolites of Artemisia plants include terpenoids, flavonoids, coumarins, glycosides, steroids, and polyacetylenes (Tan et al., 1998). The crude extract of *A. sieversiana* was shown to have insecticidal activity (Liu et al., 2010). In this regard, we also found that the treatment with *A. sieversiana* alone significantly reduced the mortality of *O. asiatica*. Correspondingly, it caused a series of immune and physiological responses in the host insect after *M. anisopliae* infected the host (Wang et al., 2013; Ling et al., 2016). Therefore, the high concentration of conidia may enhance host fungal infections and cause high mortality in a relatively short time (Kirkland et al., 2004; Shrestha et al., 2015). This study also indirectly confirmed that a higher concentration of *M. anisopliae* enhances its lethality.

Fungal insecticides have good potential for pest control, as there is currently no record of development of insect resistance against them (Behie and Bidochka, 2013; Erler and Ates, 2015). In order to reduce the risk of resistance, the use of fungal agents should be minimized. The efficacy of a combination of fungi and chemicals was shown to be higher under laboratory or field conditions, and has been reported in several studies involving *Anomala cuprea* Hope and *Locusta migratoria* (Hiromori and Nishigaki, 1998; Jia et al., 2016). Although there was no significant difference between the mortality of *O. asiatica* treated with 20% crude extract of *A. sieversiana* combined with 10^8^ spores/g *M. anisopliae* and 10^8^ spores/g *M. anisopliae* alone in this study, the overall performance of *O. asiatica* was decreased. This indicates that the efficacy of the crude extract of *A. sieversiana* was significantly improved.

The death time of pest is an important factor affecting the control efficacy of insecticides for pest management (Asi et al., 2008; Gupta et al., 2009). The results of a field application of 6 × 10^7^ spore *M. anisopliae* bait on locusts, showed that the peak infection of the locust population was reached on the 15th day after infection, with a mortality rate of 6.5%, and the mortality rate increased with time (Dong et al., 2011). Compared to this experiment, the mortality rate was much lower than that in the treatment with crude extract of *A. sieversiana* and *M. anisopliae*, and the peak time of infection was three times longer than that under the mixture of crude extract of *A. sieversiana* and *M. anisopliae*. The results showed that treatment with the combination of the crude extract of *A. sieversiana* and *M. anisopliae* significantly shortened the lethal time, and its efficacy was significantly better than that of other groups and any single agent. This shows that the synergetic control effect from the combination treatment was faster and better than the control effect of each pesticide alone. The effect of 10% *A. sieversiana* crude extract mixed with 10^7^ spores/g *M. anisopliae* on the mortality of *O. asiatica* reached the effect of 10^8^ spores/g *M. anisopliae* alone in this study; therefore, the dosage of *M. anisopliae* was reduced, and may largely prevent the development of resistance in insects against it.

Although it has not been reported that insects resistance to *M. anisopliae* (Dubovskiy et al., 2013), the risk of resistance to *M. anisopliae* can be reduced by observing the effects of *A. sieversiana* and *M. anisopliae* on the protective enzyme activities of *O. asiatica*. We found that the enzymatic activities of catalase and polyphenol oxidase of *O. asiatica* were over-expressed under the mixed treatment of the combination of the crude extract of *A. sieversiana* and *M. anisopliae*. And some studies have found that the high expression of catalase could shorten the germination time and increase the pathogenicity. of *M. anisopliae* (Hernandez et al., 2010). Therefore, we found that the crude extract of *A. sieversiana* can be used as a synergist of *M. anisopliae* to improve its pathogenicity. Under the conditions of external factors, such as plants and biological pesticides, the slower growth and development of insects are often associated with the expression of enzyme activity in their bodies (Serebrov et al., 2006). Our results also confirmed the conclusion that the mortality, body weight, growth rate, and overall performance of *O. asiatica* were significantly reduced under the treatment with crude extract of *A. sieversiana* and *M. anisopliae*, while the activity of its protective enzymes was significantly increased.

The use of *M. anisopliae* in combination with chemical agents have caused varied mortality rates of locusts, and even showed antagonistic effect (Jia et al., 2016). In our research, similar results of different concentration ratios causing varied mortality of *O. asiatica* were recorded. Therefore, in the considerations for the use of a combination of new biopesticides and fungal preparations for pest control, the compatibility of the two should be carefully evaluated. A number of studies have comprehensively evaluated the compatibility of fungi and chemicals agents under laboratory and field conditions (Cuthbertson et al., 2005; Asi et al., 2010).

We found an interesting phenomenon in this study. *Metarhizium anisopliae* can promote the increase of body weight and body length of *O. asiatica*. However, when *M. anisopliae* was mixed with the crude extract of *A. sieversiana*, it inhibited the growth of body weight and length, especially the growth of body weight, and the effect of the mixture of *M. anisopliae* and the crude extract of *A. sieversiana* on the development of female was greater than that of male. However, the reasons for this phenomenon need to be further studied. Our work was carried out under laboratory conditions. We, therefore, propose that further studies under field conditions characterized by many complicated and uncontrollable factors, be carried out to determine the best compound formula which shows promising efficacy. Additionally, other parameters such as the toxicity of fungi, the influence of the mixture of crude extract of *A. sieversiana* and *M. anisopliae* on natural enemies and non-target organisms, etc. should be considered in the evaluation of the combination of fungi and new biopesticides.

In summary, our research showed that *M. anisopliae* was a good biological control agent for on *O. asiatica*. The crude extract of *A. sieversiana* increased the virulence of *M. anisopliae* and shortened the mortality time of the pest. These results indicate that the crude extract of *A. sieversiana* could be used as a synergist for *M. anisopliae*, and this compound formulation can become an alternative biological control agent to control insect pest.

## Data Availability Statement

The original contributions presented in the study are included in the article/supplementary material, further inquiries can be directed to the corresponding authors.

## Author Contributions

ZZ and XT designed the experiments. SL and XT performed the experiments. SL and ZZ wrote the manuscript. SL, CX, and GD collected the data and analyzed it. ZZ and GW provided technical and material support. All authors contributed to the article and approved the submitted version.

### Conflict of Interest

The authors declare that the research was conducted in the absence of any commercial or financial relationships that could be construed as a potential conflict of interest.
